# Manipulation of Cell:Cell Contacts and Mesoderm Suppressing Activity Direct Lineage Choice from Pluripotent Primitive Ectoderm-Like Cells in Culture

**DOI:** 10.1371/journal.pone.0005579

**Published:** 2009-05-18

**Authors:** James N. Hughes, Jennifer M. Washington, Zhiqiang Zheng, Xiuwen K. Lau, Charlotte Yap, Peter D. Rathjen, Joy Rathjen

**Affiliations:** 1 School of Molecular and Biomedical Science, The University of Adelaide, Adelaide, South Australia, Australia; 2 The Australian Stem Cell Centre Monash University, Clayton, Victoria, Australia; 3 The Australian Research Council Special Research Centre for the Molecular Genetics of Development, The University of Adelaide, Adelaide, South Australia, Australia; 4 Department of Zoology, University of Melbourne, Parkville, Victoria, Australia; Katholieke Universiteit Leuven, Belgium

## Abstract

In the mammal, the pluripotent cells of embryo differentiate and commit to either the mesoderm/endoderm lineages or the ectoderm lineage during gastrulation. In culture, the ability to direct lineage choice from pluripotent cells into the mesoderm/endoderm or ectoderm lineages will enable the development of technologies for the formation of highly enriched or homogenous populations of cells. Here we show that manipulation of cell:cell contact and a mesoderm suppressing activity in culture affects the outcome of pluripotent cell differentiation and when both variables are manipulated appropriately they can direct differentiation to either the mesoderm or ectoderm lineage. The disruption of cell:cell contacts and removal of a mesoderm suppressor activity results in the differentiation of pluripotent, primitive ectoderm-like cells to the mesoderm lineage, while maintenance of cell:cell contacts and inclusion, within the culture medium, of a mesoderm suppressing activity results in the formation of near homogenous populations of ectoderm. Understanding the contribution of these variables in lineage choice provides a framework for the development of directed differentiation protocols that result in the formation of specific cell populations from pluripotent cells in culture.

## Introduction

At gastrulation in the mammal, pluripotent cells of the epiblast, or primitive ectoderm, lose pluripotency and commit to either the mesoderm/endoderm lineages or the ectoderm lineage. In the embryo, these events are spatially separated and occur in response to discrete signaling environments established in the anterior or posterior regions of the gastrula. The ability to recapitulate these events *in vitro* during pluripotent cell differentiation would enable directed differentiation technologies and the formation of highly enriched populations of normal, functional cells that can be used as research tools, as reagents in pharmacological trials and potentially as cellular adjuncts for the treatment of human disease. Moreover, recapitulation of a particular differentiation pathway *in vitro* would provide an accessible model to study the formation and subsequent differentiation of cellular intermediates.

Embryonic stem cells were first isolated from the pluripotent cells of the inner cell mass of the mouse blastocyst [1], [2] and retain many of the properties of this population in culture [3], [4]. In comparison with embryonic development, these cells represent a population of pluripotent cells morphologically and genetically distinct from the primitive ectoderm. ES cells have been used widely as a model to understand the molecular regulation of lineage establishment from pluripotent cells in culture and by extrapolation in the embryo [5]. However, the use of ES cells to model molecular events at and around gastrulation is limited by the initial and spontaneous formation of extraembryonic endoderm concurrent with the establishment of a primitive ectoderm-like cell [6], [7]. Extraembryonic endoderm acts as a source of endogenous signaling molecules that regulate further differentiation from the pluripotent cells thereby confounding the interpretation of the actions of exogenously added molecules. Considerable success has been achieved with the purification of differentiating cells from ES cell-based differentiation models and subsequent manipulation in culture to define immediate post-gastrulation events [8]. This approach, however, still relies on the spontaneous formation of a primitive ectoderm-like population from ES cells and subsequent lineage determination.

Early primitive ectoderm-like (EPL) cells are an *in vitro* model of the primitive ectoderm that can be formed without the concomitant formation of the extraembryonic endoderm [9]–[11]. EPL cells are formed from ES cells in response to the conditioned medium, MEDII, and share characteristic gene expression, differentiation potential and cytokine responses with the primitive ectoderm [9], [12], [13]. MEDII conditioned medium is derived from a human hepatocellular carcinoma cell line, HepG2 cells, and has been shown to contain distinct bioactivities responsible for the formation of a primitive ectoderm-like cell in culture [9], [14]. Subsequent differentiation of EPL cells in culture can be manipulated to form either near homogenous populations of neurectoderm without the formation of mesoderm [15] or populations deficient in neurectoderm and highly enriched in mesoderm [13]. Differentiation of EPL cells to the ectoderm lineage defaults to the neural lineage and does not appear to form populations representative of epidermal ectoderm, as shown by the lack of expression of *cytokeratin 8*, *cytokeratin 18* or *desmoplakin* within the system (JR unpublished). The establishment of neurectoderm or mesoderm to the exclusion of the alternate outcome suggests that the manipulations used in these differentiation methodologies act to alter lineage choice from differentiating EPL cells.

The differentiation of EPL cells to neurectoderm occurs in cellular aggregates in which cell:cell contacts are maintained in the presence of the conditioned medium MEDII [15]. In contrast, the enrichment of mesoderm to the exclusion of neurectoderm occurs from EPL cells that have been physically dissociated and removed from MEDII [13]. Here we determine the respective roles of cell:cell contact and MEDII in lineage choice; we show that the effects of the two manipulations are additive and that single lineage outcomes can only be achieved when both variables are manipulated appropriately. MEDII acts to impose an ectoderm fate on differentiating cells by suppressing the formation of mesoderm, even in the presence of the mesoderm-inductive activities in serum. This activity is not specific to MEDII but can be substituted by antagonists of TGF-β signaling. Disruption of cell:cell contact promotes the formation of mesoderm, and we speculate that the loss of cell:cell contact during mesoderm formation in the primitive streak may function to ensure the loss of pluripotence and spatially correct lineage choice. Understanding the contribution of these variables in lineage choice provides a framework for the development of directed differentiation protocols that can be applied to the formation of specific cell populations from pluripotent cells in culture.

## Materials and Methods

### Cell culture

The D3 ES cell line [16] and *Mixl1:GFP* ES cell line [17](kindly provided by Dr. A. Elefanty, Monash University, Australia) were used in this study. The routine culture of ES cells, formation of EPL cells and the production of MEDII conditioned medium were as described previously [11]. EPL cells formed in suspension culture for 3 days were used [9], [11] and where indicated were reduced to single cell suspension using Trypsin-EDTA (0.5% Trypsin/5.3 mM EDTA), enzyme-free Dissociation buffer (Invitrogen) or Dispase II neutral protease (Roche). Prior to trituration aggregates were incubated for 1 minute at RT with Trypsin-EDTA, for 15 minutes at 37°C with Dissociation buffer or 30 minutes at 37°C with Dispase (1 mg/mL). Suspensions were passed through a 70 µm sieve (Falcon) to remove any residual clumps. Embryoid bodies formed from dissociated EPL cells (MEDII^−^/Dis^+^EBs and MEDII^+^/Dis^+^EBs; Dis^+^ = dissociated) were initiated by seeding cells at a density of 1×10^5^ cells/mL in non-adherent bacterial petri dishes.

The differentiation potential of cells within aggregates was analyzed by seeding aggregates onto gelatin-treated tissue culture grade plastic ware (Falcon) for approximately 12 hours before the medium was replaced with chemically defined medium [11]. Outgrowths were examined microscopically 2, 4 and 6 days after seeding and scored for the presence of neural projections, visible red blood cells and beating cardiocytes. Cells with neural projections have been shown previously to express Tubulin-βIII and NeuN [15]. Generally, for each experimental condition 48 individual wells were seeded with randomly selected cellular aggregates and experiments were repeated three times. Data was analyzed statistically using a two-tailed student's t-test.

### Separation of medium

#### Ultrafiltration

Serum free MEDII (sfMEDII) was prepared and fractionated as described in Rathjen et al. [9] with the following modifications: Phenol red free DMEM (Gibco) was used as the base medium and medium was supplemented with 110 gm/L sodium pyruvate. Alternatively, serum-containing MEDII was used for the purification of eluate (E). HepG2 cells show greater viability in serum containing media so this was the preferred medium for subsequent MEDII production and medium fractionation (data not shown). Medium fractions prepared from media containing serum, but not conditioned by HepG2 cells, showed no activity within our bioassay suggesting that detected activity was derived from the HepG2 cells (data not shown). MEDII was passed over an Amicon YM3 ultrafiltration membrane in a 200 mL Amicon stirred cell under nitrogen pressure as described in [9]. The eluated (E) and retained (R) fractions were collected, reconstituted in ES cell incomplete media and filter sterilized through a 0.22 µm syringe filter (Sartorius) before use in tissue culture assays. E was supplemented with 10% FCS and used at 50% volume. The volume (mL) of R used for each mL of culture medium was calculated as 50% of the total volume of R collected after ultrafiltration/the total volume MEDII subjected to ultrafiltration. For example, if 1 mL of R is obtained from 100 mL MEDII, then 50 µL was added to 10 mL of medium.

#### Superdex peptide column

E was freeze dried, resuspended in H_2_O and filtered through a 0.22 µm filter prior to loading onto a Superdex® peptide column HR 10/30 (Pharmacia) on a Pharmacia FPLC machine with LKB controller. The sample was eluted in H_2_O and 1 mL fractions were collected. Fractions were freeze-dried, resuspended in an appropriate volume of media and filter sterilized prior to cell culture assay.

### H&E stained sections

Cell aggregates were washed in PBS, fixed in 4% PFA for 30 minutes, washed twice in PBS and embedded in paraffin wax. 5 µm microtome sections were affixed to Poly-L-Lysine coated slide (ProSciTech), dewaxed using Histoclear, rehydrated through an ethanol/H_2_O gradient and stained with hematoxylin and eosin (H&E).

### Flow cytometry

Cellular aggregates to be analyzed by flow cytometry were reduced to single cell suspensions using Trypsin-EDTA as described above and passed through a 70 µm sieve (Falcon). Cell suspensions were analysed using a Becton Dickinson FACScan and data collected using CellQuest Pro software (Becton Dickinson) and manipulated using either CellQuest Pro or FCS Express (Microsoft). D3 ES cell-derived populations were used to determine background fluorescence at each time point.

### Gene Expression Analysis

#### Real Time PCR (qRT-PCR)

Total cytoplasmic RNA was isolated using Trizol reagent (Invitrogen). cDNA was synthesized from total RNA with the Omniscript RT kit (Qiagen). The PCR reaction mix comprised 12 µL SYBR Green PCR Reaction mix (Invitrogen), 25 ng cDNA and 200 nM of each primer made up to a total volume of 25 µL with water. Samples were heated to 50°C for 2 minutes, then 95°C for 10 minutes, before being cycled 40 times through 95°C for 15 seconds and 56°C for 1 minute on an MJ research thermocycler with a Chromo4 Continuous Fluorescence Detection system (MJ Research). The raw data was analyzed using the Q-Gene software package [18], [19]. Unless otherwise indicated primers were designed using Primer3 software [20]. The sequences and length of amplified products were:

*BMP4* (118 bp) 5′ AGGAGGAGGAGGAAGAGCAG 3′

 5′CCTGGGATGTTCTCCAGATG 3′

*brachyury* (143 bp) 5′ TGCTGCCTGTGAGTCATAAC 3′

 5′ GCCTCGAAAGAACTGAGCTC 3′ [21]

*Cdh1* (67 bp) 5′ CATTTTGCAACCAAGAAAGGACT 3′

 5′ GGTTATCCGCGAGCTTGAGATGG 3′

*Cdh2* (162 bp) 5′ AGGACCTTTCCTCAAGAGC 3′

 5′ CGATCCAGAGGCTTTGTGAC 3′

*Fgf8* (143 bp) 5′ TCCGGACCTACCAGCTCTAC 3′

 5′ TCGGACTCTGCTTCCAAAAG 3′

*Mixl1* (145 bp) 5′ CTTCCGACAGACCATGTACCC 3′

 5′ GATAAGGGCTGAAATGACTTCCC 3′

*Ptn* (78 bp) 5′ TGGAGCTGAGTGCAAGTACC 3′

 5′ CTGCCAGTTCTGGTCTTCAA 3′

*Snail1* (64 bp) 5′ GCCGGAAGCCCAACTATAGC 3′

 5′ TAGGGCTGCTGGAAGGTGAA 3′

*Snail2* (117 bp) 5′ GCTCCTTCCTGGTCAAGAAACA 3′

 5′ TGACAGGTATAGGGTAACTTTCATAGAGA 3′

*Sox1* (171 bp) 5′ GACTTGCAGGCTATGTACAACATC 3′

 5′ CCTCTCAGACGGTGGAGTTATATT 3′

*Wnt3* (138 bp) 5′ TCCACTGGTGCTGCTATGTC 3′

 5′ CCTGCTTCTCATGGGACTTC 3′

*β-actin* (89 bp): 5′ CTGCCTGACGGCCAGG 3′

 5′ GATTCCATACCCAAGAAGGAAGG 3′

## Results

### Regulation of the culture media and cell:cell junctions impose lineage choice on EPL cells in culture

**Figure 1:**
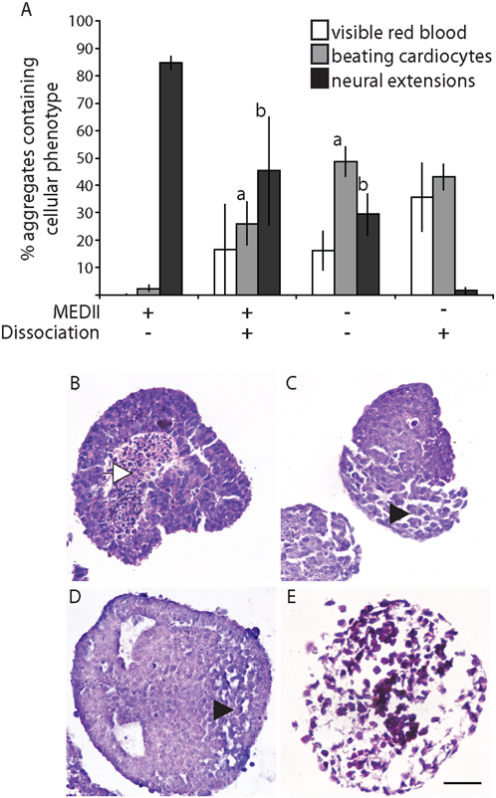
Cell dissociation and MEDII both impact on lineage choice during EPL cell differentiation. (A). EPL cells were formed in aggregates in MEDII for three days before being dissociated and reaggregated in unsupplemented tissue culture medium or medium supplemented with 50% MEDII (MEDII+). Alternatively, EPL cells were maintained in aggregates and transferred to unsupplemented tissue culture medium or medium supplemented with 50% MEDII. Aggregates were maintained for a further 4 days (to day 7) in suspension culture before being seeded individually into 48 tissue culture wells in unsupplemented medium and allowed to differentiate. Aggregates were scored for the presence of visible red blood cells, beating cardiocytes and neural extensions and the peak score for each represented. n≥3 independent experiments. Error bars represent standard error of the mean (sem). (a) denotes an increase compared to MEDII^+^/Dissociation^−^ where p<0.01. (b) denotes an increase compared to MEDII^−^/Dissociation^+^ where p<0.05. (B–D). Aggregates cultured as for A were collected and fixed on day 7 before paraffin embedding and sectioning. Sections were stained with haemotoxylin/eosin and viewed with a Ziess Axiophot microscope. (B). MEDII^+^/Dis^−^EB. (C). MEDII^+^/Dis^+^EB. (D). MEDII^−^/Dis^−^EB. (E). MEDII^−^/Dis^+^EB. Scale bar represents 50 µm. The white arrowhead in B indicates an area of cell necrosis. The black arrowheads in C and D denote areas of cell morphology similar to that found in E.

The manipulation of EPL cells during differentiation to mesoderm or neurectoderm differs with respect to two variables. When cells are differentiated to mesoderm cell:cell contacts are disrupted and MEDII is removed [13]. Conversely, efficient differentiation to neurectoderm occurs in the presence of MEDII and when cell:cell contacts are maintained [15]. EPL cells were formed in suspension culture in medium supplemented with 50% MEDII for 3 days and either reduced to single cells or left intact and cultured in the presence or absence of MEDII. The effect of these manipulations on subsequent differentiation was analyzed to determine the contribution of each variable to lineage choice during EPL cell differentiation (Figure 1A). As expected, EPL cells maintained in MEDII for a further 4 days, and without cell dissociation (MEDII^+^/Dis^−^EBs), resulted in 85% of the aggregates forming neural extensions and 2.5% of aggregates forming detectable mesoderm. The converse manipulation, where EPL cells were dissociated and reaggregated in the absence of MEDII (MEDII^−^/Dis^+^EBs) resulted in the expected formation of mesoderm, detected as visible red blood (36%) or beating cardiocytes (43%) [13]. Very few of these aggregates contained neural extensions (1.7%). Differentiation outcomes from EPL cells removed from MEDII without dissociation (MEDII^−^/Dis^−^EBs), or from aggregates formed from dissociated EPL cells aggregated in the presence of MEDII (MEDII^+^/Dis^+^EBs), were mixed, with both conditions giving rise to an intermediate number of aggregates forming detectable mesoderm or neural extensions.

The morphology of cell aggregates on day 7 reflected the observed differentiation outcomes (Figure 1B–E). Sectioning of control aggregates (MEDII^+^/Dis^−^EBs; B), showed them to be formed of densely packed cells which in areas are arranged within a columnar epithelium, consistent with the formation of neurectoderm [15]. In contrast, embryoid bodies that gave rise to outcomes enriched in mesoderm but deficient in neurectoderm, MEDII^−^/Dis^+^EBs (E), were comprised of loosely adherent, mesenchymal-like cells. MEDII^−^/Dis^−^EBs (C) and MEDII^+^/Dis^+^EBs (D) showed a mixed cell phenotype, with areas of mesenchymal-like cells adjacent to areas of densely packed cells.

These data suggest that regulation of cell:cell contacts and exposure to MEDII are capable of effecting lineage choice from EPL cells in culture.

### Gene expression analysis of differentiating EPL cells suggests a role for cell dissociation in mesoderm formation and a role for MEDII in the promotion of the ectoderm fate

**Figure 2:**
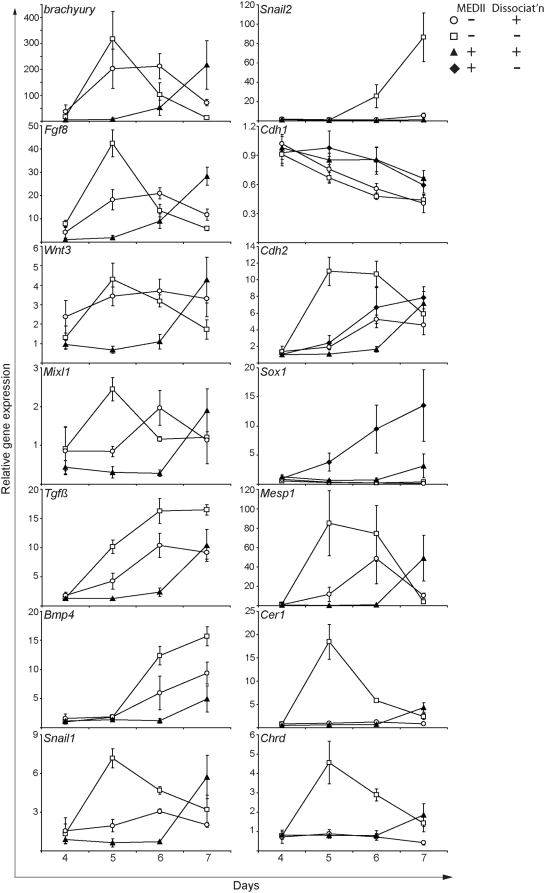
Disruption of cell:cell contact promotes the establishment of the mesoderm lineage while MEDII has an opposing activity that promotes formation of neurectoderm. EPL cells were dissociated and reaggregated in unsupplemented tissue culture medium (○) or medium supplemented with 50% MEDII (▴). Alternatively, EPL cells were maintained in aggregates and transferred to unsupplemented tissue culture medium (□) or medium supplemented with 50% MEDII (♦). Aggregates were collected daily to day 7 and analyzed by real-time PCR for the expression of markers of primitive streak, EMT and differentiated lineages. Gene expression was normalized to *β-actin* (here) and *GAPDH* (data not shown); the relative gene expression seen with either housekeeping gene was comparable. n = 4 independent experiments, error bars represent sem. MEDII^+^/Dis^−^EBs were only included where changes in gene expression were detected.

EPL cells differentiated with and without MEDII and with or without dissociation were collected every 24 hours for 4 days and analyzed for the expression of markers of the early primitive streak (*Wnt3*, *Fgf8*, *brachyury* and *Mixl1*; [22]–[26]), epithelial to mesenchyme transition (EMT) (*Snail1*, *Snail2*; [27], [28]), mesoderm (*BMP4*, *TGFβ1*; [29], [30]), E-cadherin and N-cadherin (*Cdh1*, *Cdh2*; [31]) and neural progenitors (*Sox1*; [32]) (Figure 2). Removal of MEDII resulted in the up regulation of primitive streak markers within 24 hours with expression peaking approximately 48 hours after MEDII withdrawal. Comparison of aggregates that were removed from MEDII, MEDII^−^/Dis^−^EBs and MEDII^−^/Dis^+^EBs, suggested that maintaining cell:cell contacts on withdrawal of MEDII had little effect on the timing of gene expression Primitive streak marker expression was followed by the up regulation of the differentiation markers *Cdh2*, *Tgfβ1* and *BMP4* and the concomitant down regulation of *Cdh1*. The expression of *Sox1* was not detected in aggregates which had been withdrawn from MEDII, but, as expected was detected in MEDII^+^/Dis^−^EBs, and to a lesser extent MEDII^+^/Dis^+^EBs, during this differentiation period. The expression of *Snail1* peaked 48 hours after MEDII withdrawal in MEDII^−^/Dis^−^EBs EPL cells. In comparison, this gene was poorly expressed in MEDII^−^/Dis^+^EBs. Similarly, *Snail2* expression followed *Snail1* in MEDII^−^/Dis^−^EBs but was not detected in MEDII^−^/Dis^+^EBs.

The effect of dissociation was seen when MEDII^+^/Dis^−^EBs and MEDII^+^/Dis^+^EBs were compared. Dissociated and reaggregated EPL cells differentiated in the presence of MEDII up regulated primitive streak, EMT and differentiation markers. In contrast, expression of these markers was not detected in MEDII^+^/Dis^−^EBs (data not shown).

Comparison of MEDII^+^/Dis^+^EBs and MEDII^−^/Dis^+^EBs, which demonstrates the effect of MEDII on the differentiation of cells after dissociation, suggested that MEDII delayed the up regulation of markers of the primitive streak, EMT and differentiation by 24 to 48 hours. Together these data suggest that the disruption of cell:cell contact promotes the establishment of the mesoderm lineage and that MEDII has an opposing activity that suppresses the formation of this lineage in culture.

The expression of genes specifically expressed in the anterior (*Cer1*, *Chrd*) and posterior (*Mesp1*) mesoderm were examined [33]. Expression of all three genes was detected in MEDII^−^/Dis^−^EBs, suggesting a diverse range of mesoderm lineages formed on MEDII withdrawal. In contrast, MEDII^−^/Dis^+^EBs failed to up regulate the anterior mesoderm markers suggesting the preferential formation of posterior mesoderm in these aggregates. Dissociation in the presence of MEDII also appeared to favor the formation of posterior mesoderm.

### Multiple methods of cell dissociation suppress the formation of neurectoderm from EPL cells

**Figure 3:**
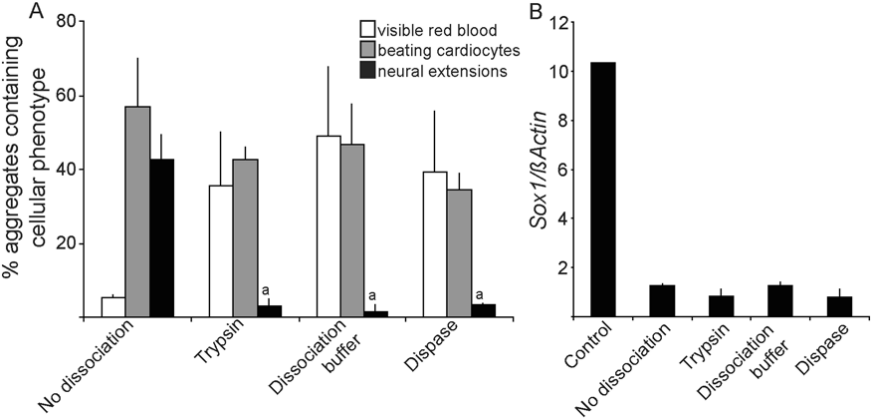
Dissociation, and not specific actions of Trypsin-EDTA, acts to suppress neurectoderm formation. (A). EPL cells, formed in aggregates in MEDII, were dissociated with Dispase, Dissociation buffer or Trypsin-EDTA, reaggregated and maintained for a further 4 days before being seeded individually into 48 tissue culture wells and allowed to differentiate. Aggregates were scored for the presence of beating cardiocytes and neural extensions and the peak score for each represented. Outcomes are compared to outcomes from EPL cells that were maintained in aggregates. n = 3 independent experiments. Error bars represent sem. (a) denotes a decrease compared to MEDII^−^/Dissociation^−^ where p<0.01. (B). EPL cells, formed in aggregates in MEDII, were either maintained in medium supplemented with 50% MEDII (control), cultured in unsupplemented medium (no addition) or dissociated with Dispase, Dissociation buffer or Trypsin-EDTA, reaggregated and maintained in suspension culture in unsupplemented medium for a further 6 days. Aggregates were collected and extracted RNA was analyzed for the presence of *Sox1*, and *GAPDH* by real-time PCR. n = 3 independent experiments. Error bars represent sem.

The effect of cell dissociation by trypsin-EDTA could be mediated through the physical breaking of cell:cell contacts or via specific effects of trypsin and/or EDTA. To distinguish between these possibilities, EPL cells were dissociated with a bacillus-derived neutral metalloprotease (Dispase, Roche), an enzyme-free dissociation buffer (Cell Dissociation Buffer, Invitrogen) or the serine protease trypsin-EDTA (0.5% Trypsin (Invitrogen)/5.3 mM EDTA), reaggregated and allowed to differentiate without MEDII (Figure 3). Differentiation outcomes from each condition were alike, with the formation of few detectable neural extensions and robust formation of visible red blood and beating cardiocytes. Similarly, we were unable to detect differences in the expression of *Sox1* in aggregates formed after the different dissociation methods (Figure 3B). These data suggest that the physical breaking of cell:cell contacts, rather than trypsin or EDTA mediated signaling, is regulating lineage choice. Surprisingly, we were not able to detect higher levels of *Sox1* expression in MEDII^−^/Dis^−^EBs in comparison to MEDII^−^/Dis^+^ EBs, despite the robust formation of neurons from these aggregates, suggesting that the transient nature of the neural progenitor in MEDII^−^/Dis^−^EBs made detection of the progenitor difficult.

### A mesoderm-suppressing activity in MEDII

**Figure 4:**
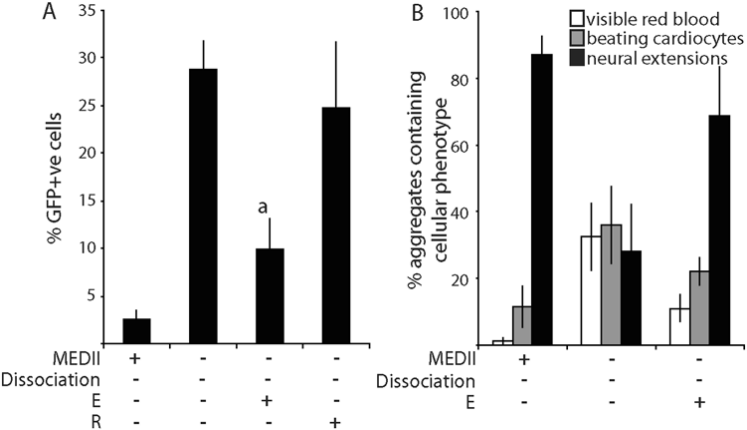
The MEDII-associated mesoderm suppressing activity is found within the small molecular weight components of the medium. (A). *Mixl1:GFP* ES cells were cultured as aggregates in MEDII for 3 days to form EPL cells. Aggregates were transferred to unsupplemented medium or medium supplemented with 50% MEDII, 50% E or R and cultured for a further 2 days before reduction to a single cell suspension. The proportion of GFP^+^ cells present was determined by flow cytometry. n = 7 independent experiments (MEDII^−^Dis^−^R^+^ n = 3). Error bars represent sem. (a) represents a decrease when compared with MEDII-Dis- where p<0.001. (B). EPL cells were prepared as for (A) and transferred to unsupplemented medium or medium supplemented with 50% MEDII or 50% E. Aggregates were maintained in culture for a further 4 days before being seeded individually into 48 tissue culture wells and allowed to differentiate. Aggregates were scored for the presence of visible red blood cells, beating cardiocytes and neural extensions and the peak score for each represented. n = 3 independent experiments and includes data generated from both *Mixl1:GFP* and D3 ES cell lines. Error bars represent sem.

The lack of mesoderm in MEDII^+^/Dis^−^EBs, and the delay in gene expression changes associated with mesoderm formation in MEDII^+^/Dis^+^EBs compared to MEDII^−^/Dis^+^EBs, suggests that MEDII contains an activity that suppresses the formation of mesoderm from EPL cells. MEDII was fractionated by ultrafiltration through a 3×10^3^ *M_r_* cut-off membrane (Centricon-3 unit, Amicon; [9] to give a retained fraction (R) and an eluted fraction (E). EPL cells were formed from ES cells with GFP knocked-in to one allele of the *Mixl1* locus, allowing the identification of early mesoderm by flow cytometry (*Mixl1:GFP* ES cells; [17]). From day 3, EPL cells were maintained in culture without dissociation in unsupplemented medium (MEDII^−^/Dis^−^EBs), or in medium supplemented with E (50%), R (equivalent to 50%MEDII) or MEDII (MEDII^+^/Dis^−^EBs). Cell aggregates were allowed to differentiate for a further two days before analysis for the presence of GFP positive cells (Figure 4). As expected, the removal of MEDII from EPL cells resulted in an up regulation of *Mixl1* expression reflected in GFP expression in approaching 30% of the cells. A similar level of GFP expression was detected in aggregates cultured in medium supplemented with R. In contrast, significantly fewer GFP^+^ cells were detected in aggregates cultured in the presence of E (10%; p = 0.0012) suggesting that the mesoderm suppressive activity is contained within this fraction.

*Mixl1:GFP* ES cell-derived EPL cells maintained in aggregates and cultured in medium supplemented with MEDII, 50% E or unsupplemented medium were seeded on day 7 and allowed to differentiate (Figure 4B). Compared to cells cultured in unsupplemented medium, fewer aggregates cultured in 50% E formed mesoderm while a greater number formed neural extensions, consistent with a suppression of mesoderm formation and concomitant enrichment of neurectoderm.

**Figure 5:**
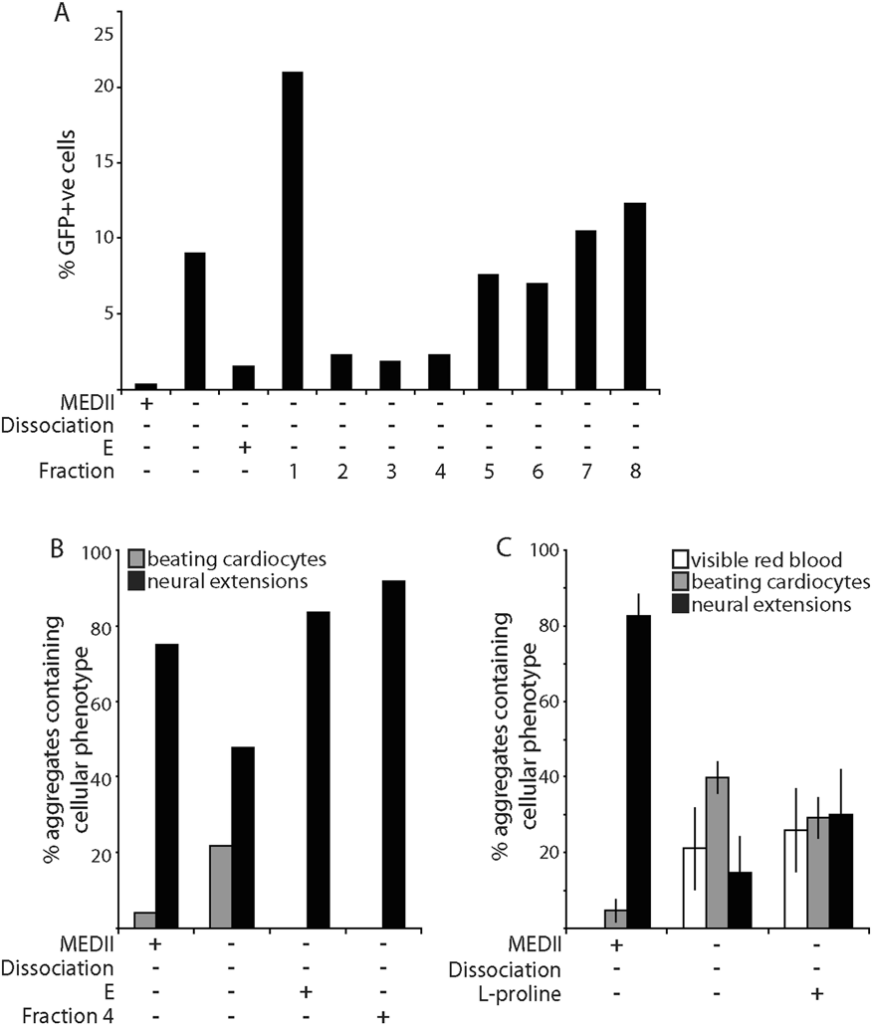
The MEDII-associated mesoderm suppressing activity is a discrete moiety within the medium. (A). E was fractionated over a Superdex® peptide column. *Mixl1:GFP* ES cells were cultured as aggregates in MEDII for 3 days to form EPL cells. Aggregates were transferred to unsupplemented medium or medium supplemented with 50% MEDII, 50% E or a 50% MEDII equivalent volume of fractions 1–8 and cultured for a further 2 days before reduction to a single cell suspension. The proportion of GFP^+^ cells present was determined by flow cytometry. (B). EPL cells were prepared as for (A) and transferred to unsupplemented medium or medium supplemented with 50% MEDII, 50% E or fraction 4. Aggregates were maintained in culture for a further 4 days before being seeded individually into 48 tissue culture wells and allowed to differentiate. Aggregates were scored for the presence of visible red blood cells, beating cardiocytes and neural extensions and the peak score for each represented. (C). EPL cell aggregates were formed from *Mixl1:GFP* ES cells for 3 days in MEDII supplemented medium before being transferred to unsupplemented medium or medium supplemented with 50% MEDII or 200 µM l-proline. Aggregates were maintained in culture for a further 4 days before being seeded individually into 48 tissue culture wells and allowed to differentiate. Aggregates were scored for the presence of visible red blood cells, beating cardiocytes and neural extensions and the peak score for each represented. n = 3 independent experiments. Error bars represent sem.

The mesoderm suppressor activity within E was not retained on a Sephadex® G25 matrix (Sigma-Aldrich), suggesting that the activity was smaller than 1 kDa (data not shown). E was fractionated on a Superdex® peptide FPLC column (Pharmacia) and fractions tested for their ability to suppress the formation of GFP^+^ cells from EPL cells derived from *Mixl1:GFP* ES cells (Figure 5A). Addition of fractions 2–4 to differentiating EPL cells reduced the formation of GFP^+^ cells to levels comparable to EPL cells differentiated in medium supplemented with 50% E. Further analysis of fraction 4 showed that this fraction contained an activity which reduced the number of aggregates forming beating cardiocytes and enhanced the number of aggregates containing neural extensions (Figure 5B). Comparison of the elution profile of fractions 2–4 with molecular weight standards suggests that the activity has a molecular weight of between 100 and 1000 Da.

We have previously identified a small bioactive molecule, l-proline, in the eluate of serum free MEDII with the ability to induce the differentiation of ES cells in culture ([9]; JW unpublished). Addition of 200 µM l-proline (Sigma-Aldrich) to MEDII^−^/Dis^−^EBs did not suppress the formation of mesoderm (Figure 5C) suggesting that the mesoderm suppressor activity was distinct from l-proline.

### Modulation of signaling by TGFβ family members regulates mesoderm formation from EPL cells in culture

**Figure 6:**
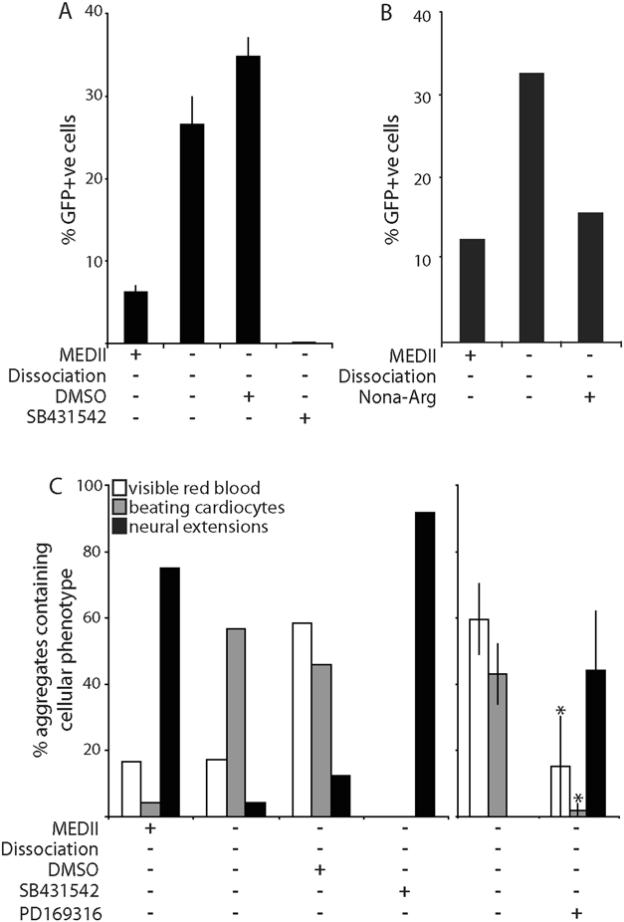
Tgf-β antagonists suppress mesoderm formation from EPL cells in culture. *Mixl1:GFP* ES cells were cultured as aggregates in MEDII for 3 days to form EPL cells. Aggregates were transferred to unsupplemented medium or medium supplemented with 50% MEDII, 0.4% DMSO or 10 µM SB431542 (prepared in DMSO) and cultured for a further 2 days before reduction to a single cell suspension. The proportion of GFP^+^ cells present was determined by flow cytometry. n = 3 independent experiments. Error bars represent standard error of the mean. (B). EPL cells were prepared as for (A) and transferred to unsupplemented medium or medium supplemented with 50% MEDII or 77 µM Nona-Arg (prepared in H_2_0) and cultured for a further 2 days before reduction to a single cell suspension. The proportion of GFP^+^ cells present was determined by flow cytometry. A representative result is shown. (C). EPL cell aggregates were formed from ES cells and transferred to unsupplemented medium or medium supplemented with MEDII, DMSO, 10 µM SB431542 or 10 µM PD169316 as indicated. Aggregates were maintained in culture for a further 4 days before seeding them individually into 48 tissue culture wells and allowing them to differentiate. Aggregates were scored for the presence of visible red blood cells, beating cardiocytes and neural extensions and the peak score for each represented. n = 3 independent experiments (PD169316). Error bars represent sem. A representative experiment is shown for SB431542.

Signaling by TGFβ family members, notably Nodal [34] and BMP4 [29], have been implicated in the establishment of the primitive streak and formation of mesoderm during mouse gastrulation. Two small molecule antagonists of TGFβ protein activation were added to *Mixl1:GFP* ES cell-derived MEDII^−^/Dis^−^EBs to determine the involvement of TGFβ signaling in mesoderm induction from EPL cells in culture (Figure 6 A,B). The peptide, nona-L-arginine, that has been shown to inhibit the enzymatic activity of the Subtilase-like Pro-Protein Convertases (SPC) protease Furin [35], and SB431542 (Sigma-Aldrich), a chemical inhibitor of the Alk4, Alk5 and Alk7 receptors, were used in this analysis. Addition of either antagonist reduced the formation of GFP^+^ cells during EPL cell differentiation in comparison to untreated MEDII^−^/Dis^−^EBs suggesting that functional TGFβ signaling is required for *Mixl1* expression from EPL cells.

EPL cells were differentiated in the presence of SB431542 or PD169316 (Sigma-Aldrich) (Figure 6C). PD169316 has been shown to inhibit Smad2 phosphorylation in human ovarian cancer cells and ES cells ([36]; CY unpublished). Differentiation assays suggested that the loss of primitive streak marker gene expression that occurs in response to inhibition of TGFβ signaling was accompanied by a subsequent reduction in mesoderm formation and an increase in the number of aggregates containing neural extensions.

## Discussion

### Roles for cell:cell contact and MEDII in lineage choice

Comparing the outcome from EPL cells differentiated after dissociation/reaggregation in the absence of MEDII with EPL cells differentiated as aggregates in MEDII suggests that the differentiation of EPL cells is directed to a specific outcome by the culture manipulations. Here we have undertaken to determine the relative importance of cell:cell contact and components within MEDII in the determination of lineage choice from primitive ectoderm-like cells in culture. Manipulation of these relatively simple parameters in combination efficiently directed cells to adopt either mesoderm or ectoderm fates.

The effects of cell dissociation and MEDII on lineage choice were opposing, with dissociation promoting the formation of posterior fates/mesoderm and MEDII favoring the formation of anterior fates/ectoderm. Neither variable, however, was completely dominant, with mesoderm and neurectoderm forming in the presence of MEDII from EPL cells that had been dissociated or from cells that had been maintained as aggregates but from which MEDII had been withdrawn. The ability of dissociated EPL cells to respond to MEDII, or of undissociated cells to form mesoderm on MEDII withdrawal, suggests that as cells differentiate they maintain a degree of developmental plasticity that allows them to gauge multiple environmental cues before committing to a cellular outcome. Previous studies and data presented here suggest that the combined effect of dissociation with withdrawal of MEDII, or maintenance of cell:cell contact and MEDII, is to direct EPL cell differentiation almost exclusively to a single lineage, mesoderm and neurectoderm respectively [13], [15].

### The role of cell dissociation in mesoderm determination from primitive ectoderm-like cells: formation of a cell committed to differentiation

Within the embryo, specification and determination of mesoderm and endoderm in the primitive streak are regulated by BMPs, Nodal and Wnts (reviewed in [37]) and accompanied by an epithelial to mesenchymal transition. Cells deficient in *Fgfr1* fail to form mesoderm at the primitive streak but form a secondary neural tube, a phenotype that has been postulated to result from an inability to undergo an EMT [38] and suggesting a requirement for the EMT in mesoderm determination. The expression of *Snail1* and *Snail2* in EPL cells as they differentiate to mesoderm infers that these cells undergo an EMT during differentiation. It is tempting to speculate that the enforced loss of cell:cell contact between EPL cells is equivalent to an EMT, explaining the role of cell:cell dissociation in promoting mesoderm formation seen here. Gene expression analysis of differentiating cells, however, suggests that EMT-specific genes appear between 24 and 48 hours after dissociation suggesting that dissociation and EMT are distinct events. The up regulation of the EMT-specific genes is accompanied by morphological alterations consistent with a loosening of cell adhesion within the aggregates and adoption of a mesenchymal-like phenotype (data not shown).

Dissociation of cell:cell contact here appears to be acting to initiate differentiation and promote the acquisition of a transitory cell state in which the cells are primed to differentiate but not yet specified to either cell lineage. Specification of mesoderm from these cells is likely to occur from signals contained within the serum or endogenously generated signals. The differentiation of EPL cells in serum free medium significantly reduces the formation of mesoderm, implicating serum as a source of mesoderm inducing activities in this system ([39]; data not shown). It is interesting to note that the earliest markers of mesoderm specification detected were *Wnt3* and *Fgf8*. Although no role for Fgf8 in mesoderm specification has been determined, Wnt3 has been well-documented as required for the specification of mesoderm within the primitive streak of the gastrulating mouse embryo [22] and in culture [8], [33], [40] and may be playing an analogous role within these cell aggregates.

The complete loss of cell:cell contact does not occur during pluripotent cell differentiation in the embryo, hence the significance of this event to differentiation appears to be specific to differentiation in culture. Data presented here suggests that cell dissociation acts to promote differentiation towards mesoderm, preferentially promoting the formation of more posterior mesoderm. The dissociation of *Xenopus laevis* animal caps similarly demonstrates the importance of cell:cell contact in cell fate choice and the consequences of disruption of cell:cell contact can have on further differentiation [41]. It is interesting to note that many differentiation protocols and technologies for cellular analysis require cell dissociation but have failed to recognize the potent bioactivity associated with this event (for example [42]).

### The role of mesoderm suppression in promoting ectoderm formation

The data presented here are consistent with the presence of a discrete mesoderm suppressing activity in MEDII that acts to oppose mesoderm induction from primitive ectoderm-like cells in culture. Estimations of the size of the MEDII-derived activity, between 100 and 1000 Da, suggest that the suppressor activity is a novel moiety (or moieties), distinct from activities that regulate mesoderm-promoting activities *in vivo*, such as *Noggin*, *Chordin* and *Dickkopf1* [43]–[45]. The small size of the MEDII-derived activity does not exclude the presence of a small peptide inhibitor of the TGF-β processing enzymes [35].

*Nodal^−/−^* embryos exhibit a loss of mesoderm and elaboration of ectoderm during gastrulation, suggesting that Nodal signaling is required for mesoderm formation from the primitive ectoderm [46]. Inhibition of TGF-β signaling during EPL cell differentiation resulted in a loss of mesoderm formation similar to that seen with MEDII. Similarly, *Wnt^−/−^* embryos exhibit a loss of mesoderm at gastrulation [22] and inhibition of Wnt signaling by Dkk1 in differentiating ES cells inhibited the formation of mesoderm lineages [40]. This suggests that other activities capable of suppressing mesoderm formation can substitute for the activity of MEDII in the regulation of lineage choice from EPL cells and is consistent with the implication of multiple pathway inhibitors in the suppression of mesoderm on the anterior of the gastrulating embryo [47].

The imposition of lineage commitment on differentiating pluripotent cells in culture has been a long-term challenge for ES cell biologists. Here we describe culture manipulations that can effectively direct differentiation of ES cells to a specific germ lineage in culture. The ability to direct the differentiation of pluripotent cells into progenitors of a single germ lineage is predicted to have several ramifications on further differentiation. The number of terminally differentiated cell populations that can be formed when compared to spontaneously differentiated pluripotent cells in culture will be reduced. A reduction in cellular complexity will in turn decrease the complexity of the endogenous signaling environment and the diversity of inductive cell:cell interactions that can occur. This will result in greater opportunities to direct further differentiation through the addition of exogenous factors. Directing differentiation is also likely to reduce the need for sophisticated cell sorting technologies to achieve enriched cell populations for research and potentially clinical applications. Cues that direct primitive ectoderm-like cells to alternate lineages, as described here, could usefully be incorporated with defined regimes for the induction of mesoderm subtypes [33], or for formation of neural progenitors [48] for the formation of populations of somatic cells in culture.
